# Retinal Response to Low-Level Red-Light Therapy in Myopic and Non-Myopic Eyes Assessed Using Global Flash Multifocal Electroretinogram

**DOI:** 10.3390/vision10030037

**Published:** 2026-06-27

**Authors:** Muhammad Qasim, Paulo Fernandes, Jorge Jorge

**Affiliations:** Physics Center for Universities of Minho and Porto (CF-UM-UP), School of Sciences, University of Minho, Gualtar Campus, 4710-057 Braga, Portugal; jorge@fisica.uminho.pt

**Keywords:** global flash multifocal ERG, red-light therapy, myope, non-myope, retina

## Abstract

**Purpose**: This study was conducted to assess retinal responses in myopic and non-myopic participants after exposure to repeated low-level red-light therapy (RLRT) of 3 min and 1 min duration to see if response differs with exposure time and refractive status using global flash multifocal electroretinogram (gf-mfERG). **Methods**: This was a prospective exploratory pilot study with a convenient sample of 20 participants of mean age of 25.5 years (18 to 39 years) who underwent 3 min therapy, followed by, 1 week after, 1 min therapy in each participant between February to May 2025. However, four participants who did not appear in 1 min therapy for measurements were excluded from the between-group comparison analysis. We measured retinal responses using gf-mfERG ring-wise implicit time (DCT, ICT) and amplitude (DCA, ICA), baseline, post-first and post-second RLRT. Data analysis included descriptives, repeated measures ANOVA, post hoc test, Bonferroni adjusted correction, and correlation with axial length (AL) and choroidal thickness (CT) in each group. The study was conducted at CEORLab, University of Minho, Portugal, and adhered to Ethical Committee approval and the tenets of the Declaration of Helsinki. **Results**: In 3 min RLRT exposure, no significant within-subject changes were observed across direct (DC) and induced (IC) gf-mfERG components across all rings (*p* > 0.05). Statistically significant between-group differences in DC implicit time were noted in Ring 2 (*p* = 0.029) and Ring 3 (*p* = 0.012). IC amplitude showed significant within-subject changes in myopes (Ring 1: *p* = 0.030; Ring 4: *p* = 0.035) and non-myopes (Ring 1: *p* = 0.042; Ring 3: *p* = 0.001; Ring 4: *p* < 0.001; Ring 5: *p* = 0.034), with no significant between-group differences (*p* > 0.05). In contrast, 1 min RLRT showed no significant changes in DC, IC time or amplitudes ICA, DCA (all *p* > 0.05). Correlation analysis after 3 min RLRT showed that in myopes, DCT positively correlated with AL in Ring 3 (r = 0.591, *p* = 0.006) and Ring 4 (r = 0.615, *p* = 0.004), ICT positively correlated with CT in Ring 2 (r = 0.601, *p* = 0.005) and Ring 3 (r = 0.573, *p* = 0.008), and ICA negatively correlated with AL in Rings 3–5 (all *p* < 0.01). Non-myopes demonstrated limited correlations, including a negative association between ICT and axial length in Ring 4 (r = −0.619, *p* = 0.004). Similar but weaker patterns were observed following 1 min RLRT. **Conclusions**: 3-min RLRT induces localized inner retinal functional changes without significantly altering outer retinal responses, while 1-min exposure shows no measurable effect. Retinal ERG responses are primarily influenced by axial length, particularly in myopic eyes, highlighting a structure–function relationship rather than a robust short-term therapeutic effect of RLRT.

## 1. Introduction

Historically, myopia management focused largely on correcting vision with spectacles or contact lenses. In recent decades, strategies have been shifted towards interventions aimed at slowing myopia progression and reducing the risk of long-term ocular complications associated with high myopia [1]. These contemporary approaches include optical treatments such as orthokeratology and multifocal soft contact lenses [2], pharmacological interventions like low-dose atropine [3], and behavioral modifications such as increasing time spent outdoors and reducing near work [4], or non-invasive approaches like low-level red-light therapy [5]. Despite these advances, further research is required to better understand the mechanisms underlying these interventions and to evaluate their long-term efficacy. In particular, objective measures capable of detecting retinal functional changes may provide important insights into treatment effects beyond subjective visual outcomes [6].

The human electroretinogram (ERG) has been an important tool in evaluating retinal function since its introduction in the early 20th century [7]. Vision begins when light rays are focused on to the retina, where phototransduction converts them into electrical signals that are transmitted through retinal neurons to the brain, enabling visual perception [8]. Because structural imaging alone may not reveal early functional alterations, electrophysiological techniques are valuable for detecting retinal dysfunction before clinical detectable structural changes occur [9]. Different ERG modalities assess distinct components of retinal function. The full-field ERG (ffERG) measures global retinal responses and is widely used to evaluate generalized retinal disorders [10]. Pattern ERG (PERG) primarily reflects macular and retinal ganglion cell activity and is useful for the early detection of optic neuropathies such as glaucoma [11]; multifocal ERG (mfERG) allows the assessment of localized retinal responses, particularly within the macular region, and is therefore valuable for identifying early macular dysfunction [12,13]. The global flash multifocal ERG (gf-mfERG) further enhances this approach by combining local stimulation with full-field “global” flashes, enabling separation of outer (direct component, DC) and inner (induced component, IC) retinal activity in terms of implicit time (DCT, ICT) and amplitude (DCA, ICA) [14,15,16].

Retinal and choroidal alterations associated with myopia are not always detectable through structural imaging, particularly in cases of early or moderate myopia. Electrophysiological techniques can help detect functional abnormalities before clinically observable structural changes become evident [9,10]. Electrophysiological studies using conventional mfERG have shown reduced N1 and P1 amplitudes in high myopes, indicating outer retinal dysfunction, particularly in evaluating subclinical dysfunction before visible pathology emerges [17,18]. These findings suggest that retinal dysfunction in myopia primarily involves photoreceptors and bipolar cell activity, with possible secondary involvement of inner retinal layers. Previous studies have also reported an inverse relationship between axial length and retinal response amplitudes, particularly affecting the induced component (IC), which reflects inner retinal activity. Increased axial length has been associated with reduced IC amplitudes and prolonged response latencies [16,19].

Recently, low-level red-light therapy has emerged as a non-invasive approach for myopia control. Although its exact mechanism of action remains unclear, growing evidence suggests that red-light exposure may influence retinal and choroidal structures. Experimental and clinical studies have indicated that the choroidal vasculature plays a key role in providing metabolic support to the retina and may be involved in regulating ocular growth [20]. Red-light therapy has been associated with changes in sub-foveal choroidal thickness, which may influence the positioning of the retina relative to the focal plane and potentially affect retinal signaling pathways involved in ocular growth regulation. Recent clinical studies have reported increases in sub-foveal choroidal thickness following red-light therapy, accompanied by reductions in axial length after short treatment periods [21]. Additional investigations have also demonstrated increase in choroidal and choriocapillaris luminal areas following red-light stimulation [22]. Moreover, scleral remodeling mediated by changes in collagen and extra-cellular matrix synthesis of fibroblasts potentially play a central role in axial elongation in myopia [23].

Structural and functional alterations in the inner retina, as well as changes observed in retinal electrophysiology, are strongly associated with myopia development [24]. In this context, ERG has become a valuable tool in myopia research for evaluating retinal function and monitoring treatment-related changes [25]. In this context, the prospective exploratory pilot study aims to investigate short-term retinal responses to low-level red-light therapy (RLRT) in myopic and non-myopic eyes with the primary objective of retinal functional modulation (specifically the direct and induced components of the global flash mfERG), and that this functional modulation varies depending on both exposure duration (1 vs. 3 min) and the baseline refractive state of the subjects (myopic vs. non-myopic) and its underlying mechanistic pathways rather than clinical safety purpose.

## 2. Materials and Methods

**Study Design and Participants:** This was a prospective exploratory pilot study with a convenient sample of 10 myopic and 10 non-myopic adult participants for better cooperation during electrophysiological testing because of sustained fixation, which can be achieved more reliably in adults rather than children. Also, the technical feasibility of the ERG, reliability and data quality, ethical and practical considerations for participants inclusion were considered for this mechanistic investigation. Formal sample size calculation was not performed, as the study was designed to generate preliminary physiological data and explore retinal electrophysiological responses to RLRT for hypothesis generation. Participants were recruited via an online screening questionnaire (Google Forms) followed by clinical verification. Each subject underwent 3 min RLRT and then 1 min therapy with at least a 1-week gap between February to May 2025. Both 3 min and 1 min RLRT were composed of two therapy sessions (first session and second session) separated by 30 min intervals. All 20 subjects completed 3 min therapy, but only 16 completed the 1 min therapy, because four participants did not come due to their personal reasons, forcing us to continue with the available data for presentation. We could not take another sample to complete as it would violate the structure–function comparison, although both cohorts were similar but different in numbers. Only one eye per subject was tested (the more myopic eye, or the right eye in non-myopic). Informed written consent was obtained from all the study participants.

**Eligibility Criteria:** We recruited young adults between age 18–39 years of both genders. Spherical equivalent refraction of <−0.75 D for the myopic group, and within ±0.50 D for the non-myopic group having best-corrected visual acuity ≥ 0.1 LogMAR, were the main clinical verification checks for the inclusion of participants in this study. Exclusion criteria included previous ocular surgery, systemic disease with ocular impact, current ocular medication, or inability to complete electrophysiological testing in conditions like epilepsy.

**Figure 1:**
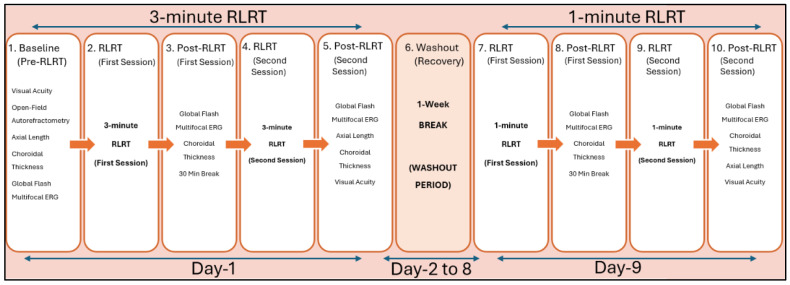
Showing sequential 3 min and 1 min RLRT protocol in myopic and non-myopic participants.

**Study Protocol:** Each participant underwent baseline measurements, which included clinical assessments (visual acuity, auto-refractometry), followed by ocular structural parameters i-e axial length (IOL Master 500, Carl Zeiss Meditec AG, Jena Germanyand choroidal thickness (MOPTIM 3000-Shenzhen Certainn Technology Co., Ltd., Shenzhen, China) and functional parameters i-e gf-mfERG electrophysiological testing (RETI-Port/Scan21, Ronald Consults, Weisbaden, Germany). Then, low-level red-light therapy (RLRT) was performed using an Eyerising Myproclear Myopia Management Device (650 ± 10 nm) (Eyerising International Pty Ltd., Melbourne, Victoria, Australia) for 3 min or 1 min therapy, separated by 30 min intervals. The fellow eye was occluded during the therapy exposure. Structural (choroidal thickness) and functional (gf-mfERG) parameters were repeated post-first therapy exposure. All the clinical (visual acuity with and without correction), structural (axial length, choroidal thickness) and functional parameters (gf-mfERG) were re-assessed post-second therapy exposure in all the participants separately at the 3 min and 1 min RLRT sessions, as shown in Figure 1. The mean average of the five consecutive readings in millimeters (mm) was recorded as an axial length measurement of each participant during assessment.

**Figure 2:**
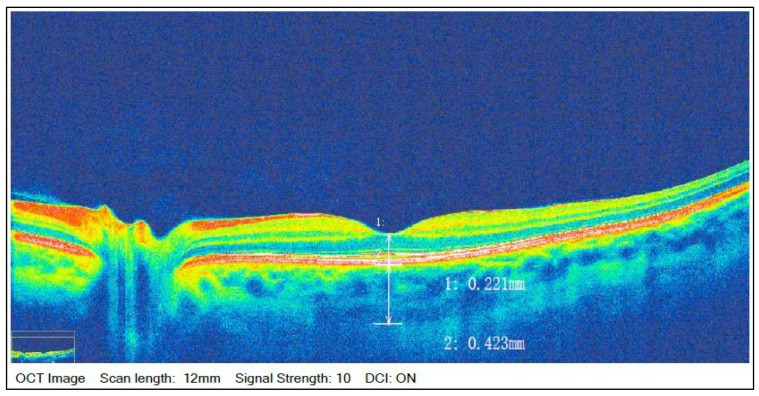
Showing deep choroidal imaging (DCI) scan obtained via Moptim OCT.

Central choroidal thickness measurement was taken using high-definition (HD) deep choroidal imaging acquisition (DCI) mode (based on scanning laser ophthalmoscopic imaging) with a line width of 12 mm for each measurement. A minimum scan signal of 8 out 10 was kept for the capturing of the image. Manual measurement procedures were carried out by the investigator down the central foveal point to the choroidal posterior extent in a perpendicular intersection, and the measurement shown in OCT scan was recorded as shown by the OCT in-built comprehensive software tool in micrometers (μm), which can be seen below in the given Figure 2.

**Figure 3:**
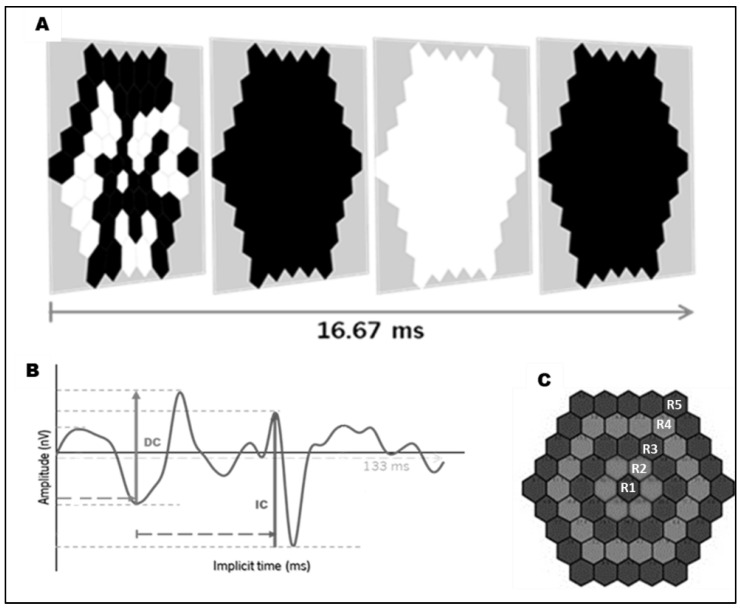
(**A**) Showing gf-mfERG bright and dark stimulation frames, (**B**) showing retinal response recording of direct (DC) and induced (IC) component; (**C**) showing ring-wise activity of gf-mfERG.

**Electrophysiology acquisition:** Retinal function was recorded following ISCEV principles. Pupils were not dilated due to the participants undergoing RLRT exposure, which is carried out in normal pupils only. Visual stimuli consisted of 61 scaled hexagonal elements presented in a pseudorandom sequence, interleaved with full-field global black-and-white flashes at a frame rate of 75 Hz (Figure 3A). Stimuli were displayed on an LCD monitor positioned at 33 cm [26]. Active DTL fiber electrodes were used for signal acquisition, with reference electrodes placed near the lateral canthus and a ground electrode positioned at the mid-forehead. Skin preparation was performed prior to electrode placement, and electrode impedance was continuously monitored and strictly maintained below 10 kΩ throughout recording in order to obtain good-quality data. The standard protocol was identical for all subjects and time points to ensure that any detected change was a true reflection of acute retinal responses to RLRT. For analysis, gf-mfERG peak time (ms) and amplitude (µV) of the direct and induced components (DC and IC, respectively) of the first-order kernel were extracted using peak-to-peak measurements (Figure 3B). DCT and ICT represented temporal parameters of direct and induced components, while DCA and ICA represented their corresponding amplitudes [12,14]. Regional analysis was performed using five concentric retinal eccentricity rings centered on the fovea (Figure 3C) [16,27]. These regions were defined as: Ring 1 (0–2°) corresponding to the fovea; Ring 2 (2–7.6°) to the parafovea; Ring 3 (7.6–14.8°) to the perifovea; Ring 4 (14.8–23°) near the peripheral retina; and Ring 5 (23–30°) in the central mid-retinal periphery [28].

**Statistical analysis:** Descriptive statistics are presented as mean ± standard deviation for the measurements taken at baseline, post-first therapy and post-second therapy. Prior to running repeated measures ANOVA, normality was systematically assessed using the Shapiro–Wilk test for each parameter (DCT, ICT, DCA, ICA) and across all ERG rings. Homogeneity of variance (homoscedasticity) was evaluated using Mauchly’s test of sphericity, applying the Greenhouse–Geisser correction whenever the assumption of sphericity was violated. We evaluated the same individual across three longitudinal time points (baseline, post-1st session, and post-2nd session), and we utilized a repeated measures ANOVA for making within-subject correlation in this data structure. Appropriate non-parametric alternatives were applied in cases where deviation from normality was violated and tests like Friedman’s test were applied to confirm that the overall statistical conclusion remain unchanged. To control multiple comparisons and avoid type I error, strict Bonferroni correction was applied to all post hoc pairwise analyses, which are thoroughly detailed in the supplementary data. Relationships between gf-mfERG parameters (DC and IC implicit times and amplitudes) and structural parameters (axial length—AL and choroidal thickness—CT) were assessed using Pearson’s correlation for normally distributed data and Spearman’s correlation for non-normally distributed data. Effect sizes and 95% confidence intervals were reported where applicable and are also provided in the Supplementary Materials. A *p*-value of <0.05 was considered statistically significant. The statistical significance observed in our results is driven by consistent intra-individual shifts in response to low-level red-light therapy, showing the robustness and reliability of the data despite the diverse baseline values. All statistical analyses were conducted using JASP version 0.96 (University of Amsterdam, Netherland).

## 3. Results

Baseline characteristics of the study participants are presented in Table 1. The mean age of participants ranged from 24.4 to 26.3 years. Myopic participants demonstrated a mean spherical equivalent of approximately −2.50 D with longer axial lengths (24.49 mm) compared to non-myopic participants (23.91 mm). Choroidal thickness was relatively comparable between groups, ranging from 367.6 μm to 370.3 μm, with similar visual acuity profiles across the cohort.

**Figure 4:**
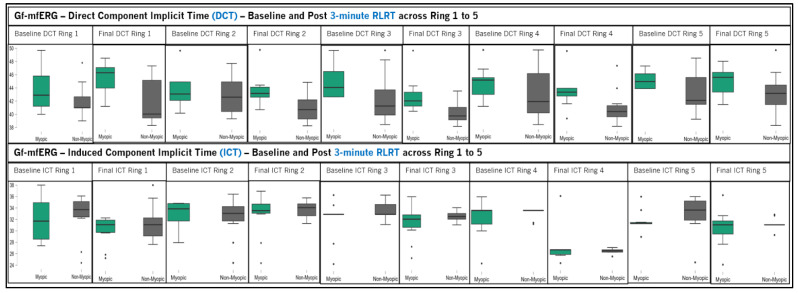
Shows gf-mfERG implicit time (DCT, ICT) in myopic and non-myopic participants before and after 3-min RLRT.

Figure 4 presents gf-mfERG implicit time (DCT and ICT) in myopic and non-myopic participants following 3 min RLRT. DCT showed no significant within-subject changes across baseline, post-first therapy and post-second therapy in myopic and non-myopic participants across all rings (*p* > 0.05). However, significant between-group differences were noted in Ring 2 (*p* = 0.029, F = 5.648, ηp^2^ = 0.239) and Ring 3 (*p* = 0.012, F = 7.777, ηp^2^ = 0.302), indicating regional differences in DCT responses between myopic and non-myopic participants after RLRT. ICT remained relatively stable across baseline and post-therapy measurements in myopic and non-myopics, with no significant within-subject effects or between-group differences across rings (*p* > 0.05).

**Figure 5:**
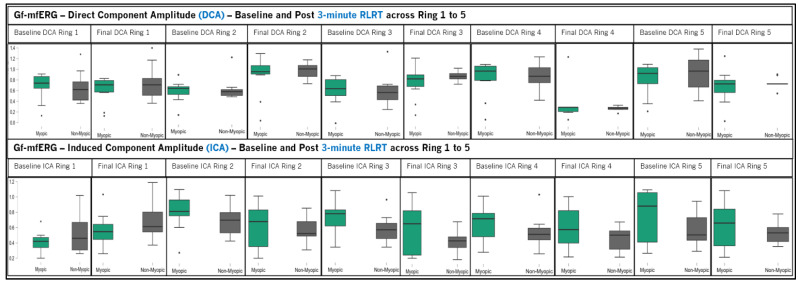
Shows gf-mfERG amplitudes (DCA, ICA) in myopic and non-myopic participants before and after 3-min RLRT.

Figure 5 presents gf-mfERG amplitudes (DCA & ICA) in myopic and non-myopic participants following 3 min RLRT. DCA values remained relatively stable across baseline, post-first therapy and post-second therapy in both myopic and non-myopic participants. DCA results showed neither significant within-subject effects nor between-group differences among myopic and non-myopic participants across all rings (*p* > 0.05). ICA showed significant within-subject changes observed in the myopic group in Ring 1 (*p* = 0.030, F = 4.296, ηp^2^ = 0.323) and Ring 4 (*p* = 0.035, F = 4.706, ηp^2^ = 0.312), while non-myopes showed significant changes in Ring 1 (*p* = 0.042, F = 3.810, ηp^2^ = 0.297), Ring 3 (*p* = 0.001, F = 9.848, ηp^2^ = 0.522), Ring 4 (*p* < 0.001, F = 20.82, ηp^2^ = 0.698), and Ring 5 (*p* = 0.034, F = 4.112, ηp^2^ = 0.314), respectively. Ring 4 and four *p*-values showed Mauchley’s test of sphericity, indicating that the assumption of asphericity was violated (*p* < 0.05), while no significant between-group difference was found in ICA responses between myopic and non-myopic participants following 3 min RLRT.

**Figure 6:**
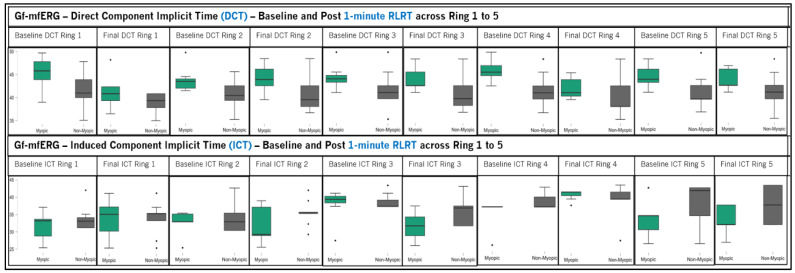
Shows gf-mfERG implicit time (DCT & ICT) in myopic and non-myopic participants before and after 1-min RLRT.

Figure 6 presents gf-mfERG implicit time (DCT & ICT) in myopic and non-myopic participants following 1 min RLRT. Neither DCT nor ICT analysis showed significant within-subject changes or between-subject group differences across baseline, post-first therapy, and then post-second therapy, across all rings (*p* > 0.05).

**Figure 7:**
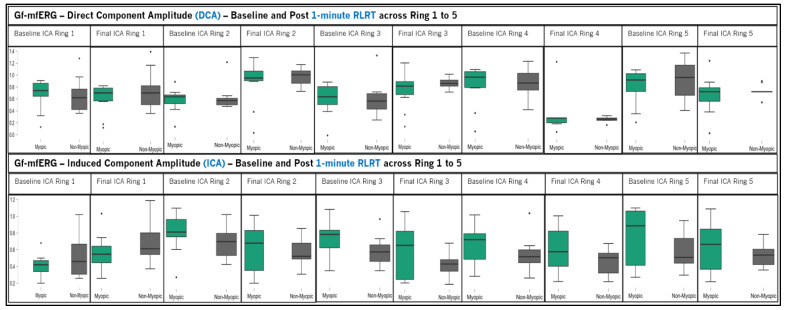
Shows gf-mfERG amplitude (DCA, ICA) in myopic and non-myopic participants before and after 1-min RLRT.

Figure 7 presents gf-mfERG amplitude (DCA and ICA) in myopic and non-myopic participants following 1 min RLRT. DCA values showed minimal variation across baseline, post-first therapy and post-second therapy in both myopic and non-myopic groups. No significant within-subject changes were observed across all rings (*p* > 0.05). ICA measurements remained relatively stable across all time points in both groups, with no significant changes detected across rings (*p* > 0.05). This indicates that 1 min RLRT did not produce significant alterations in amplitude responses in either myopic or non-myopic participants.

**Figure 8:**
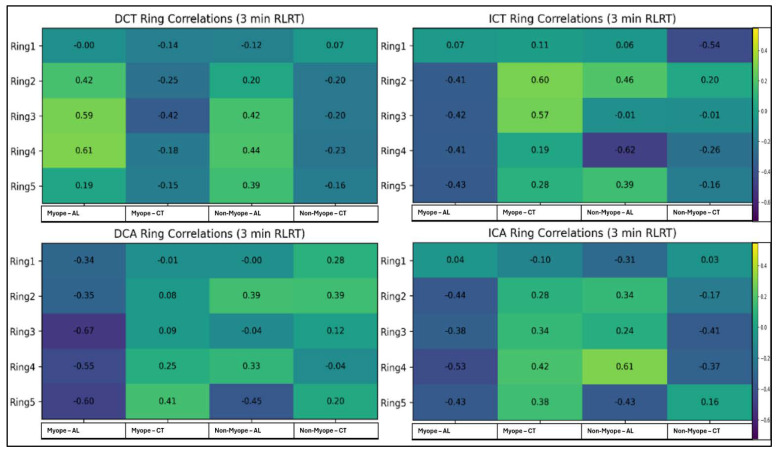
Correlation of gf-mfERG (DCT, ICT, DCA, ICA) ring-wise with AL and CT in 3 min RLRT.

Figure 8 presents correlation of gf-mfERG components (DCT, ICT, DCA, ICA) ring-wise with AL and CT following 3 min RLRT. DCT demonstrated a significant positive correlation in myopic particpants with AL in Ring 3 (r = 0.591, *p* = 0.006, F = 0.679) and Ring 4 (r = 0.615, *p* = 0.004, ES 0.717), while CT showed no significant association across any ring. In non-myopic participants, no significant correlation was found with AL or CT (*p* > 0.05). ICT showed a significant positive correlation in myopic participants only with CT in Ring 2 (r = 0.601, *p* = 0.005, F = 0.695) and Ring 3 (r = 0.573, *p* = 0.008, F = 0.653). In non-myopic participants, ICT demonstrated a significant negative correlation with AL in Ring 4 (r = −0.619, *p* = 0.004, F = −0.724). In myopes, DCA demonstrated significant negative correlations only with AL and not with CT (*p* > 0.05) in Ring 3 (r = −0.666, *p* = 0.001, F = −0.804), Ring 4 (r = −0.546, *p* = 0.013, F = −0.613) and Ring 5 (r = −0.601, *p* = 0.005, F = −0.694), while in non-myopic, only Ring 5 (r = −0.452, *p* = 0.003, F = −0.488) showed significant negative correlation with AL. In myopes, ICA showed significant negative correlations with AL only in Ring 4 (r = −0.534, *p* = 0.015, F = −0.595), while no significant correlation was observed with CT. In non-myopic, statistically significant correlations with AL were identified in Ring 4 (r = 0.614, *p* = 0.004, F = 0.715) and Ring 5 (r = −0.433, *p* = 0.005, F = −0.464). Ninety-five per cent CI or further information of all these correlation can be found in the Supplementary Materials of this article.

**Figure 9:**
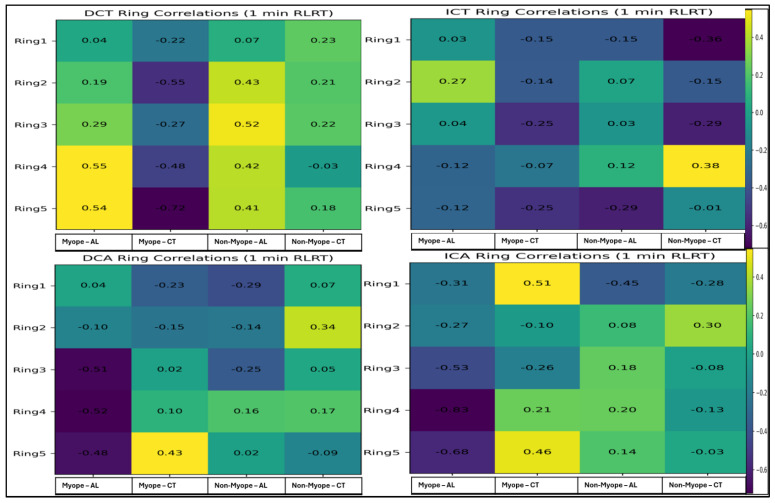
Correlation of gf-mfERG (DCT, ICT, DCA, ICA) ring-wise with AL and CT in 1 min RLRT.

Figure 9 presents correlation of gf-mfERG components (DCT, ICT, DCA, ICA) ring-wise with AL and CT following 1 min RLRT. In myopic particpants, DCT demonstrated significant correlation with AL in Ring 4 (r = 0.546, *p* = 0.043, F = 0.613) and Ring 5 (r = 0.543, *p* = 0.045, F = 0.609), while only in Ring 3 (r = 0.520, *p* = 0.027, F = 0.576) among non-myopic participants. Significant negative correlation with CT was observed among myopic in Ring 2 (r = −0.553, *p* = 0.040, F = −0.623) and Ring 5 (r = −0.723, *p* = 0.003, F = −0.915), while no significant correlation with CT was observed in non-myopic. In either group, no significant correlation of ICT was found with AL or CT (*p* > 0.05). DCA demonstrated no significant correlations with AL or CT in either group. ICA showed significant negative correlations with AL in Ring 4 (r = −0.834, *p* < 0.001, F = −1.201) and Ring 5 (r = −0.680, *p* = 0.007, F = −0.829) only in myopic subjects, while not with CT (*p* > 0.05) in either group. Ninety-five per cent CI or further information of all these correlation can be found in the Supplementary Materials of this article.

## 4. Discussion

This prospective exploratory pilot study investigated retinal electrophysiological responses after RLRT using gf-mfERG in myopic and non-myopic eyes. We calculated and analyzed the longitudinal changes (delta values) relative to each subject’s own baseline at each specific stimulation time point. The main findings can be summarized as follows: (1) RLRT did not induce significant within-subject changes in outer retinal timing, although regional between-group differences were observed following 3 min exposure; (2) inner retinal timing (ICT) remained largely stable across both treatment durations; (3) Short-term duration (1 min) did not produce measurable electrophysiological modulation as compared to 3 min exposure; and (4) retinal electrophysiological responses were significantly associated with structural ocular parameters, particularly axial length. Together, these findings suggest that RLRT reflects subtle localized electrophysiologic variability or structural–functional associations related to axial length and refractive error rather than direct therapeutic effects.

A key finding of this study is the association between retinal electrophysiological responses and ocular structural parameters. In myopic eyes, increasing axial length was associated with reduced outer retinal amplitude and delayed implicit time responses, particularly in parafoveal and peripheral regions. These findings support the concept that axial elongation is a major determinant of retinal functional modulation in myopia. Conversely, non-myopic eyes demonstrated minimal correlations between electrophysiological parameters and axial length, suggesting that physiological variations in ocular size have limited functional impact.

Choroidal thickness exhibited a distinct relationship with retinal timing parameters. Thicker choroids were associated with delayed inner retinal implicit times, while amplitude measures remained largely unaffected. This pattern may reflect the choroid’s role in outer retinal metabolic support and oxygen delivery, which can influence electrophysiological timing without directly affecting response amplitude.

Regional analysis further indicated that parafoveal and inferior retinal regions showed greater sensitivity to axial elongation. These findings are consistent with previous observations that mechanical stretching in myopic eyes is not uniformly distributed across the posterior pole.

### 4.1. Effects of RLRT on Retinal Electrophysiology

The present results indicate that RLRT primarily affects the temporal characteristics of photoreceptor-driven responses. Changes in DCT were observed across several retinal rings, particularly under short-duration stimulation. In contrast, the absence of significant alterations in ICT suggests that inner retinal processing remains resilient to RLRT exposure. This selective modulation of outer retinal timing may reflect altered retinal functional responses following low-level red-light therapy. Red wavelengths have been shown to influence mitochondrial activity and cellular metabolism, particularly through cytochrome c oxidase activation, which may modulate retinal energy dynamics and downstream visual processing pathways [29]. Clinical studies of repeated low-level red-light therapy (RLRT) in myopia have demonstrated associated changes in choroidal thickness and axial length, suggesting broader effects on ocular growth regulation [21,30]. The stability of amplitude parameters across most retinal regions in the present study further suggests that RLRT may primarily influence temporal processing of retinal responses rather than significantly altering response magnitude. Together, these findings indicate that RLRT may exert functional effects on retinal signaling dynamics, although the underlying cellular mechanisms remain to be fully elucidated.

**Implicit Time:** Historically, the peripheral and mid-peripheral retina have been shown to play a critical role in generating the visual signals that guide emmetropization and eye growth. Specifically, current evidence on myopia management [31] demonstrates that the retinal region spanning approximately 5° to 15° of eccentricity is highly sensitive to optical defocus and light stimulation, acting as a primary zone for the upregulation or downregulation of eye growth signals. In the 3 min RLRT condition, significant DCT alterations were observed in Rings 2 and 3 within the parafoveal region (representing retinal eccentricity of 2° and 15°), suggesting localized modulation of outer retinal temporal processing under prolonged red-light exposure. Similar stimulus-dependent modulation of outer retinal response timing has been described in global flash mfERG studies assessing retinal adaptation dynamics [14,16]. However, the absence of ICT changes indicates relative preservation of inner retinal timing stability, consistent with prior evidence that induced component responses are more resilient to certain forms of retinal adaptation and primarily reflect inner retinal function. These findings suggest a selective effect of RLRT on outer retinal temporal dynamics without widespread involvement of inner retinal timing mechanisms. This interpretation is consistent with established mfERG frameworks describing differential contributions of retinal layers to DC and IC components [12]. Furthermore, the stability of ICT implies that inner retinal processing remains functionally stable under short-duration RLRT exposure, indicating a preferential modulation of outer retinal response timing. Additionally, previous mfERG studies have demonstrated that retinal functional responses vary with the degree of myopia, with more pronounced electrophysiological alterations observed in higher myopia compared to low myopia and emmetropia [17,18,24].

**Amplitude:** Following 3 min RLRT, DCA amplitudes remained stable across all retinal rings in both myopic and non-myopic groups, with no significant within-subject or between-group differences observed (*p* > 0.05), suggesting that outer retinal activity was not substantially affected by the intervention. In contrast, ICA amplitudes demonstrated significant within-subject changes in selected retinal regions. Myopes showed significant changes in Rings 1 and 4 (*p* < 0.05), while non-myopes exhibited significant alterations in Rings 1, 3, 4 and 5 (*p* < 0.05). The larger number of significant changes and greater effect sizes observed in non-myopes suggest a more pronounced modulation of inner retinal function following 3 min RLRT in this group. However, the absence of significant between-group effects indicates that the overall therapy response did not differ significantly between refractive groups. Collectively, these findings suggest that 3 min RLRT duration preferentially influences ICA-related inner retinal activity, while DCA-related responses remain largely unaffected. Following 1 min RLRT, neither DCA nor ICA amplitudes demonstrated significant within-subject changes across any retinal ring in either myopic or non-myopic participants (all *p* > 0.05), and no significant between-group differences were observed. Although modest increases in DCA amplitudes were noted across most rings in myopes and slight fluctuations were evident in non-myopes, these changes did not reach statistical significance. Similarly, ICA amplitudes showed a tendency to increase across several rings in myopes, particularly after the second therapy session, whereas non-myopes exhibited minor reductions or stable responses; however, none of these changes were statistically significant. The absence of significant effects in both DCA and ICA components suggests that a 1 min RLRT exposure is insufficient to induce measurable alterations in outer or inner retinal electrophysiological activity, regardless of refractive status.

Physiologically, myopia is associated with retinal stretching, choroidal thinning, and altered photoreceptor alignment, which may influence outer retinal electrophysiological activity [32,33]. Previous studies using multifocal and global flash mfERG have demonstrated delayed implicit times and localized amplitude alterations in myopic eyes, suggesting subtle functional retinal changes rather than widespread retinal dysfunction [34]. These findings align with the previous mfERG literature demonstrating that implicit time changes often preceded amplitude reduction and serve as a sensitive marker of early functional modulation [12].

Notably, the comparable stability of inner retinal parameters between groups suggests that RLRT does not adversely affect bipolar or inner retinal layers in either refractive category. This supports the safety profile of RLRT in both myopic and non-myopic eyes, while also indicating that outer retinal elements are the primary site of measurable electrophysiological modulation.

Overall, both myopic and non-myopic eyes demonstrate preserved central electrophysiological responses following RLRT, suggesting maintained core retinal functional integrity. However, myopic eyes exhibit relatively great variability in outer retinal temporal responses, particularly under prolonged stimulation (3 min RLRT), whereas non-myopic eyes show more consistent response patterns under shorter exposure conditions. These observations suggest that refractive status may influence the dynamics of retinal temporal adaptation, although the overall integrity of retinal recovery responses appears preserved. This interpretation is consistent with previous gf-mfERG studies demonstrating that retinal adaptation responses and temporal characteristics vary with the degree of myopia [16].

### 4.2. Correlations of gf-mfERG with Axial Length and Choroidal Thickness

This study demonstrates a clear structure–function relationship in myopic eyes, highlighting the differential influence of AL and CT on gf-mfERG responses under both 3 min and 1 min RLRT. The data reveal that axial elongation is the primary determinant of retinal functional alterations, whereas CT selectively modulates inner retinal timing.

**Axial Length:** In myopic eyes, increasing axial length was strongly associated with reduced outer retinal response amplitude (DCA) and delayed outer retinal implicit time (DCT), particularly in parafoveal and peripheral retinal regions. Inner retinal amplitude (ICA) also showed a negative association with axial length, suggesting functional alterations across retinal layers with increasing ocular elongation. These electrophysiological findings indicate that axial elongation may be associated with both outer and inner retinal functional changes.

These results are consistent with structural evidence showing that high myopia is associated with retinal stretching, photoreceptor displacement, and progressive remodeling of retinal layers, including changes in the inner nuclear and outer retinal architecture [35,36].

**Choroidal Thickness:** Choroidal thickness (CT) demonstrated a distinct pattern, with evidence of associations with inner retinal implicit time (ICT), while showing minimal relationship with amplitude parameters. Increased CT in myopic eyes was associated with delayed retinal response timing, suggesting a potential link between choroidal structural status and retinal functional dynamics.

Choroid plays a critical role in ocular physiology by regulating metabolic support, oxygen diffusion, and growth signaling to the outer retina, and is considered an important modulator of retinal function during ocular development [20]. Structural studies have also demonstrated a significant inverse relationship between AL and CT, indicating progressive choroidal thinning with increasing myopia [37].

Variation in responses to myopic subjects were also observed, which shows the well-known phenomenon of heterogeneity in myopic progression and treatment responsiveness. We hypothesize that this differential behavior suggests not all myopic eyes respond to acute light therapy in the same manner or at the same rate. This could be due to several factors like scleral/choroidal remodeling differences where long-standing structural changes in more advanced myopic eyes (e.g., thinner baseline choroid or altered scleral stiffness) might dull the immediate vascular or neural response to a short stimulation. Secondly, the non-responders vs. responders, as in clinical trials of RLRT therapy [5,21] where a percentage of children show less efficacy in axial length suppression, our short-term electrophysiological data may be capturing the acute functional precursor of this distinct responsiveness.

### 4.3. Influence of RLRT Duration

All the available published studies have used 3 min RLRT as a standard therapy while there are no such studies using 1 min RLRT. This present study gives an insight into 3 min RLRT vs. 1 min RLRT. Comparison between 3 min and 1 min RLRT conditions revealed consistent correlation patterns, indicating that structural determinants, rather than stimulation duration, primarily govern electrophysiological responses. The consistency across both RLRT paradigms reinforces gf-mfERG as a reliable measure of structural–functional integrity in myopic eyes. DC and IC components of the gf-mfERG reflect different aspects of retinal function. DC is considered to originate from outer retinal activity, whereas IC is thought to reflect adaptive processes involving inner retinal circuitry. In the present study, significant changes were observed only in the DC following 3 min RLRT, suggesting that immediate retinal response to RLRT may occur primarily at the level of outer retina. In contrast, no significant changes were observed after 1 min RLRT. This may indicate that the exposure duration might be insufficient to elicit measurable physiological effects, or any induced changes were smaller than the detection capability of the current gf-mfERG protocol. This difference between 3 min and 1 min RLRT gives an excellent finding that point towards a dose-dependent threshold for red-light therapy.

### 4.4. Regional Analysis

Regional analysis highlighted greater susceptibility of parafoveal, temporal, and inferior retinal areas to axial elongation. This spatial pattern is consistent with known regional biomechanical effects of myopic eye growth, where posterior pole expansion and asymmetric scleral remodeling lead to non-uniform retinal stretching and localized structural vulnerability in high myopia [35]. Additional imaging studies have demonstrated that high myopia is associated with preferential structural alterations in the posterior and peripheral retina, supporting region-specific susceptibility to axial elongation-related changes [30].

Clinically, these findings suggest that gf-mfERG amplitude measures may serve as sensitive biomarkers for early functional retinal alterations in regionally vulnerable areas, while temporal parameters may provide additional insight into retinal response dynamics associated with axial elongation. This interpretation is supported by previous global flash mfERG studies demonstrating refractive-error-dependent changes in retinal adaptation responses and functional sensitivity across retinal regions [16].

Overall, our results confirm that axial elongation predominantly drives electrophysiological compromise, whereas CT selectively influences inner retinal timing. These insights enhance understanding of structure–function relationships in myopia and may guide functional monitoring in clinical and therapeutic contexts.

### 4.5. Limitations

A key limitation of this study is the absence of a priori sample size calculation and the relatively small cohort size of 10 myopic and 10 non-myopic participants. As an exploratory pilot study, the sample was limited to 20 participants in 3 min RLRT and 16 in 1 min RLRT, which may reduce statistical power and increase the risk of type II error. Therefore, the findings should be interpreted as preliminary and hypothesis-generating rather than confirmatory. Further, due to the high density of statistical findings, the findings that certain isolated correlations or single-ring differences represent false positive findings cannot be entirely ruled out.

In addition, participants were recruited from a single geographic region, which may restrict the generalizability of the findings to populations with different genetic backgrounds or environmental risk profiles for myopia. Furthermore, gf-mfERG recordings may be susceptible to physiological and technical variability, including fixation instability, micro-saccades, and blink-related artifacts, which could influence signal consistency despite standardization efforts. We also acknowledge that due to the pilot exploratory nature of this in-depth gf-mfERG study and limited sample size, statistically significant findings may also have resulted in false positive findings. The present study was designed to assess retinal responses following RLRT exposure; repeated recordings were obtained after intervention rather than under stable physiological conditions. Consequently, the data are not suitable for formal test–retest reliability analysis. Future studies specifically designed to evaluate repeatability are warranted to determine minimal detectable changes and the reliability characteristics of gf-mfERG parameters.

Finally, CT assessment was limited to sub-foveal measurements, which do not fully capture regional variations in choroidal morphology across the macula and peripheral retina.

### 4.6. Future Directions

Future studies should adopt longitudinal designs to better characterize how retinal responses evolve in relation to progressive axial elongation and myopia progression. Expanding gf-mfERG analysis to include broader retinal eccentricities may provide a more comprehensive functional map of region-specific retinal alterations.

Integrating multimodal imaging approaches, such as optical coherence tomography angiography, could further elucidate the relationship between choroidal perfusion, microvascular dynamics, and retinal electrophysiological behavior. Additionally, systematic evaluation of low-level red-light therapy parameters, including exposure duration, frequency, and intensity, may help optimize stimulation protocols and clarify whether RLRT exerts protective, adaptive, or modulatory effects on retinal function in myopia.

Future studies employing animal models and histological approaches may help elucidate potential alterations in retinal pigment epithelium mitochondrial activity, choroidal vascular architecture, photoreceptor integrity, and other retinal cellular pathways that could contribute to the functional responses observed following RLRT exposure.

## 5. Conclusions

In conclusion, the preliminary findings of this prospective exploratory pilot study showed a significant association between axial length and retinal electrophysiological changes in myopia, while choroidal thickness demonstrated a relationship with retinal temporal dynamics. RLRT was associated with selective alterations in outer retinal timing with preservation of inner retinal function. Overall, these findings highlight the interaction between ocular structural changes and retinal function, and support gf-mfERG as a useful tool for detecting subtle functional alterations in myopic eyes.

## Figures and Tables

**Table 1:** Baseline characteristics of the study participants.

Category	Mean Age (yrs)	Gender		Total Subjects	Logmar Visual Acuity		Spherical Equivalent (D)	Axial Length (mm)	Choroidal Thickness (μm)
		Male	Female		Unaided	Aided			
Myope	26.3	3	7	20	−0.122	−0.180	−2.52	24.49	367.6
Non-Myope	24.6	5	5		−0.168	−0.182	0.10	23.91	370.3

## Data Availability

The raw data supporting the conclusions of this article will be made available by the authors on request.

## Supplementary Materials

The following supporting information can be downloaded at: https://www.mdpi.com/article/10.3390/vision10030037/s1, Global Flash Multifocal ERG and Correlation with AL and CT Data (including Tables S1–S12). Table S1: gfmfERG Implicit Time Ring-Wise Descriptives—3 min RLRT. Table S2: gfmfERG Implicit Time Ring-Wise Descriptives—1 min RLRT. Table S3: gfmfERG Amplitude Ring-Wise Descriptives—3 min RLRT. Table S4: gfmfERG Amplitude Ring-Wise Descriptives—1 min RLRT. Table S5: DCT Ring Correlation with AL and CT—3 min RLRT. Table S6: ICT Ring Correlation with AL and CT—3 min RLRT. Table S7: DCA Ring Correlation with AL and CT—3 min RLRT. Table S8: ICA Ring Correlation with AXL and CT—3 min RLRT. Table S9: DCT Ring Correlation with AL and CT—1 min RLRT. Table S10: ICT Ring Correlation with AXL and CT—1 min RLRT. Table S11: DCA Ring Correlation with AL and CT—1 min RLRT. Table S12: ICA Ring Correlation with AXL and CT—1 min RLRT.

## Abbreviations

The following abbreviations are used in this manuscript:

RLRT	Repeated low-level red-light therapy
gf-mfERG	Global flash multifocal electroretinogram
AL	Axial length
CT	Choroidal thickness
DCT	Direct component—time
DCA	Direct component—amplitude
ES	Effect size
ICT	Induced component—time
ICA	Induced component—amplitude
