# Supplementary material for: Retinal Response to Low-Level Red-Light Therapy in Myopic and Non-Myopic Eyes Assessed Using Global Flash Multifocal Electroretinogram
Source: Vision (Basel). 2026 Jun 27;10(3):37. doi: 10.3390/vision10030037 (PMC13398201; doi:10.3390/vision10030037)
Supplement: Supplementary file 1 [file vision-10-00037-s001.zip › vision-4361592-supplementary.pdf]

Table S1: gfmfERG – Implicit Time (DCT & ICT) Ring-Wise Descriptives – 3min RLRT

DCT RING	Myope – DCT – 3min RLRT			Within-Subject Effects P-Value	Non-Myope – DCT – 3min RLRT			Within-Subject Effects P-Value	Between Subject Effects p-value
	Baseline	After 1st Therapy	After 2nd Therapy		Baseline	After 1st Therapy	After 2nd Therapy		
RING 1	43.88±3.27	42.95±2.32	44.04±2.12	NS	41.95±2.68	41.83±3.19	41.05±2.97	NS	NS
RING 2	41.55±2.77	41.93±2.93	43.14±3.63	NS	40.96±3.30	39.90±2.54	39.41±2.61	NS	0.029* (F=5.648, ηp2 = 0.239)
RING 3	40.67±1.96	40.38±2.13	41.05±3.38	NS	38.83±2.74	38.04±2.63	37.85±2.19	NS	0.012** (F=7.777, ηp2 = 0.302)
RING 4	40.53±1.68	39.99±2.11	40.59±2.19	NS	39.30±2.55	38.71±2.24	38.52±2.36	NS	NS
RING 5	42.04±1.06	41.74±2.03	41.00±1.34	NS	40.48±2.40	40.19±2.48	39.90±2.07	NS	NS
ICT RING	Myope – ICT – 3min RLRT			Within-Subject Effects P-Value	Non-Myope – ICT – 3min RLRT			Within-Subject Effects P-Value	Between Subject Effects p-value
	Baseline	After 1st Therapy	After 2nd Therapy		Baseline	After 1st Therapy	After 2nd Therapy		
RING 1	32.02±4.01	32.26±3.75	31.34±2.13	NS	32.58±4.05	33.22±2.25	32.57±2.81	NS	NS
RING 2	32.09±1.41	31.33±3.75	31.47±3.16	NS	31.70±2.01	32.38±2.78	32.29±1.19	NS	NS
RING 3	31.70±1.95	32.30±1.45	31.44±3.06	NS	32.58±0.82	32.77±1.13	32.79±0.96	NS	NS
RING 4	31.66±1.44	32.20±1.12	33.39±8.02	NS	32.01±0.40	32.02±0.89	31.63±1.04	NS	NS
RING 5	31.41±0.79	31.81±0.67	31.87±1.79	NS	31.90±1.46	31.80±0.70	32.19±0.65	NS	NS

*Post-Hoc Test: Pbonf =0.029 **Post-Hoc Test: Pbonf =0.012   ηp2 – partial η2   NS – Not significant (P>0.05)

Table S2: gfmfERG – Implicit Time (DCT & ICT) Ring-Wise Descriptives – 1min RLRT

DCT RING	Myope – DCT – 1min RLRT			Within-Subject Effects P-Value	Non-Myope – DCT – 1min RLRT			Within-Subject Effects P-Value	Between Subject Effects p-value
	Baseline	After 1st Therapy	After 2nd Therapy		Baseline	After 1st Therapy	After 2nd Therapy		
RING 1	49.73±12.93	45.84±2.51	44.19±2.44	NS	41.72±3.83	42.04±2.74	42.69±1.28	NS	0.021 (F=6.761, ηp2 = 0.326)
RING 2	46.54±11.63	42.76±2.26	42.09±1.99	NS	39.33±2.85	38.58±4.63	39.77±2.44	NS	0.025 (F=6.256, ηp2= 0.309)
RING 3	44.73±11.82	40.54±1.95	39.99±1.70	NS	38.37±2.77	37.84±2.95	38.04±2.53	NS	NS
RING 4	45.99±12.51	41.24±1.45	40.67±1.55	NS	38.36±2.39	37.49±6.22	39.78±3.24	NS	NS
RING 5	46.81±11.30	42.09±1.53	41.96±1.58	NS	40.09±2.59	40.63±2.44	40.21±2.58	NS	NS
ICT RING	Myope – ICT – 1min RLRT			Within-Subject Effects P-Value	Non-Myope – ICT – 1min RLRT			Within-Subject Effects P-Value	Between Subject Effects p-value
	Baseline	After 1st Therapy	After 2nd Therapy		Baseline	After 1st Therapy	After 2nd Therapy		
RING 1	31.51±4.00	30.24±3.08	32.46±2.86	NS	33.60±3.47	32.63±5.74	32.53±2.38	NS	NS
RING 2	33.17±1.39	32.07±2.24	32.20±1.61	NS	33.62±1.63	35.22±5.93	33.19±1.07	NS	NS
RING 3	32.47±2.37	32.21±1.79	32.19±0.80	NS	32.94±1.08	33.91±2.72	32.84±0.70	NS	NS
RING 4	31.64±1.47	31.93±1.23	31.79±0.75	NS	32.64±0.73	34.14±6.33	31.20±2.36	NS	NS
RING 5	32.06±0.69	31.21±2.26	31.50±0.73	NS	32.53±0.84	32.19±0.95	32.30±0.92	NS	NS

Table S3: gfmfERG – Amplitude (DCA & ICA) Ring-Wise Descriptives – 3min RLRT

DCA RING	Myope – DCA – 3min RLRT			Within-Subject Effects P-Value	Non-Myope – DCA – 3min RLRT			Within-Subject Effects P-Value	Between Subject Effects p-value
	Baseline	After 1st Therapy	After 2nd Therapy		Baseline	After 1st Therapy	After 2nd Therapy		
RING 1	0.68±0.26	0.62±0.28	0.65±0.33	NS	0.67±0.29	0.67±0.17	0.61±0.17	NS	NS
RING 2	0.41±0.15	0.37±0.11	0.37±0.22	NS	0.44±0.16	0.36±0.06	0.38±0.10	NS	NS
RING 3	0.36±0.12	0.34±0.13	0.32±0.16	NS	0.36±0.13	0.29±0.05	0.29±0.05	NS	NS
RING 4	0.29±0.12	0.29±0.12	0.31±0.11	NS	0.31±0.08	0.25±0.04	0.28±0.05	NS	NS
RING 5	0.29±0.11	0.29±0.09	0.27±0.14	NS	0.33±0.12	0.24±0.07	0.27±0.06	NS	NS
ICA RING	Myope – ICA – 3min RLRT			Within-Subject Effects P-Value	Non-Myope – ICA – 3min RLRT			Within-Subject Effects P-Value	Between Subject Effects p-value
	Baseline	After 1st Therapy	After 2nd Therapy		Baseline	After 1st Therapy	After 2nd Therapy		
RING 1	0.41±0.13	0.53±0.28	0.38±0.21	0.030 (F=4.296, ηp2 = 0.323)	0.54±0.27	0.51±0.34	0.50±0.26	0.042 (F=3.810, ηp2= 0.297)	NS
RING 2	0.49±0.19	0.52±0.23	0.41±0.30	NS	0.39±0.15	0.34±0.13	0.37±0.16	NS	NS
RING 3	0.32±0.13	0.33±0.17	0.28±0.23	NS	0.23±0.11	0.16±0.10	0.17±0.10	0.001 (F=9.848, ηp2= 0.522)	NS

STRICTLY CONFIDENTIAL

RING 4	0.24±0.12	0.22±0.12	0.21±0.14	0.035 (F=4.706, ηp2= 0.312)	0.18±0.11	0.12±0.07	0.13±0.08	<0.001[a] (F=20.82, ηp2= 0.698)	NS
RING 5	0.27±0.14	0.28±0.15	0.25±0.18	NS	0.21±0.09	0.18±0.06	0.20±0.08	0.034[a] (F=4.112, ηp2= 0.314)	NS

*Baseline vs 2nd RLRT [a] Mauchley's test of sphericity indicates that the assumption of asphericity is violated ($P<0.05$)

Table S4: gfmfERG – Amplitude (DCA & ICA) Ring-Wise Descriptives – 1min RLRT

DCA RING	Myope – DCA – 1min RLRT			Within-Subject Effects P-Value	Non-Myope – DCA – 1min RLRT			Within-Subject Effects P-Value	Between Subject Effects p-value
	Baseline	After 1st Therapy	After 2nd Therapy		Baseline	After 1st Therapy	After 2nd Therapy		
RING 1	0.68±0.23	0.74±0.32	0.71±0.22	NS	0.65±0.19	0.62±0.12	0.64±0.21	NS	NS
RING 2	0.36±0.14	0.42±0.30	0.43±0.12	NS	0.40±0.09	0.43±0.08	0.38±0.15	NS	NS
RING 3	0.32±0.11	0.35±0.19	0.35±0.11	NS	0.34±0.09	0.31±0.08	0.31±0.10	NS	NS
RING 4	0.28±0.09	0.29±0.12	0.32±0.11	NS	0.27±0.08	0.27±0.09	0.26±0.08	NS	NS
RING 5	0.26±0.06	0.28±0.16	0.29±0.09	NS	0.28±0.06	0.28±0.12	0.30±0.11	NS	NS
ICA RING	Myope – ICA – 1min RLRT			Within-Subject Effects P-Value	Non-Myope – ICA – 1min RLRT			Within-Subject Effects P-Value	Between Subject Effects p-value
	Baseline	After 1st Therapy	After 2nd Therapy		Baseline	After 1st Therapy	After 2nd Therapy		
RING 1	0.34±0.20	0.37±0.22	0.60±0.43	NS	0.67±0.31	0.55±0.19	0.50±0.22	NS	NS
RING 2	0.41±0.22	0.48±0.12	0.49±0.13	NS	0.46±0.13	0.41±0.18	0.46±0.13	NS	NS
RING 3	0.19±0.09	0.23±0.08	0.23±0.08	NS	0.20±0.06	0.19±0.11	0.19±0.11	NS	NS
RING 4	0.19±0.09	0.23±0.08	0.24±0.12	NS	0.20±0.06	0.19±0.11	0.19±0.08	NS	NS
RING 5	0.19±0.09	0.22±0.07	0.26±0.10	NS	0.25±0.05	0.20±0.14	0.24±0.06	NS	NS

Table S5: Ring Correlation of DCT gfmfERG with AXL & CT in 3min RLRT among myopes & non-myopes

Ring Wise Correlation with AXL & CT	Myope					Non-Myope				
	r-value	p-value	95% CI		Effect Size	r-value	p-value	95% CI		Effect Size
			Lower	Upper				Lower	Upper	
DCT Ring 1 – AXL	-0.001*	0.997	-0.443	0.442	-0.001	-0.123*	0.607	-0.536	0.338	-0.123
DCT Ring 1 – CT	-0.135*	0.569	-0.545	0.327	-0.136	0.065*	0.787	-0.389	0.493	0.065
DCT Ring 2 – AXL	0.419**	0.066	-0.028	0.727	0.447	0.197*	0.405	-0.269	0.588	0.200
DCT Ring 2 – CT	-0.249**	0.291	-0.623	0.218	-0.254	-0.203*	0.390	-0.495	0.387	-0.067
DCT Ring 3 – AXL	0.591**	0.006	0.201	0.819	0.679	0.416*	0.068	-0.330	0.725	0.442
DCT Ring 3 – CT	-0.417**	0.068	-0.725	0.032	-0.443	-0.203*	0.391	-0.592	0.263	-0.206
DCT Ring 4 – AXL	0.615**	0.004	0.237	0.831	0.717	0.441*	0.052	-0.002	0.739	0.473
DCT Ring 4 – CT	-0.180**	0.448	-0.577	0.285	-0.182	-0.225*	0.340	-0.607	0.241	-0.229
DCT Ring 5 – AXL	0.186**	0.432	-0.279	0.581	0.188	0.390*	0.089	-0.064	0.710	0.412
DCT Ring 5 – CT	-0.152**	0.524	-0.557	0.312	-0.153	-0.160*	0.778	-0.495	0.387	-0.067

*Pearson's Correlation **Spearman's rho Correlation

Table S6: Ring Correlation of ICT gfmfERG with AXL & CT in 3min RLRT among myopes & non-myopes

Ring Wise Correlation with AXL & CT	Myope					Non-Myope				
	r-value	p-value	95% CI		Effect Size	r-value	p-value	95% CI		Effect Size
			Lower	Upper				Lower	Upper	
ICT Ring 1 – AXL	0.071*	0.767	-0.384	0.498	0.071	0.056**	0.814	-0.396	0.487	0.056
ICT Ring 1 – CT	0.112*	0.637	-0.347	0.529	0.113	-0.537**	0.015	-0.792	-0.600	0.245
ICT Ring 2 – AXL	-0.408**	0.074	-0.720	0.043	-0.433	0.456*	0.043	0.017	0.748	0.493
ICT Ring 2 – CT	0.601**	0.005	0.217	0.825	0.695	0.197*	0.405	-0.269	0.588	0.199
ICT Ring 3 – AXL	-0.420**	0.065	-0.727	0.028	-0.448	–0.007*	0.976	-0.448	0.437	-0.007
ICT Ring 3 – CT	0.573**	0.008	0.175	0.810	0.653	-0.014*	0.954	-0.454	0.431	-0.014
ICT Ring 4 – AXL	-0.414**	0.070	-0.724	0.035	-0.440	-0.619*	0.004	-0.833	-0.243	-0.724

STRICTLY CONFIDENTIAL

ICT Ring 4 – CT	0.187**	0.430	-0.279	0.581	0.189	-0.255*	0.279	-0.627	0.212	-0.260
ICT Ring 5 – AXL	-0.431**	0.058	-0.733	0.015	-0.461	0.390*	0.089	-0.064	0.710	0.412
ICT Ring 5 – CT	0.275**	0.241	-0.191	0.640	0.282	-0.160*	0.499	-0.563	0.304	-0.162

*Pearson's Correlation **Spearman's rho Correlation

Table S7: Ring Correlation of DCA gfmfERG with AXL & CT in 3min RLRT among myopes & non-myopes

Ring Wise Correlation with AXL & CT	Myope					Non-Myope				
	r-value	p-value	95% CI		Effect Size	r-value	p-value	95% CI		Effect Size
			Lower	Upper				Lower	Upper	
DCA Ring 1 – AXL	-0.335*	0.149	-0.677	0.127	-0.348	-7.547x10^{-4}**	0.997	-0.443	0.442	-7.547x10^{-4}
DCA Ring 1 – CT	-0.006*	0.979	-0.448	0.437	-0.006	0.277**	0.237	-0.188	0.641	0.285
DCA Ring 2 – AXL	-0.352*	0.128	-0.687	0.108	-0.367	0.392**	0.088	-0.061	0.711	0.414
DCA Ring 2 – CT	0.083*	0.728	-0.373	0.507	0.083	0.385**	0.094	-0.069	0.707	0.406
DCA Ring 3 – AXL	-0.666*	0.001	-0.856	-0.317	-0.804	-0.040**	0.867	-0.474	0.410	-0.040
DCA Ring 3 – CT	0.085*	0.721	-0.371	0.508	0.085	0.121**	0.613	-0.340	0.535	0.121
DCA Ring 4 – AXL	-0.546**	0.013	-0.796	-0.137	-0.613	0.325*	0.163	-0.138	0.671	0.337
DCA Ring 4 – CT	0.249**	0.290	-0.217	-0.217	0.623	-0.035*	0.884	-0.138	0.671	0.337
DCA Ring 5 – AXL	-0.601**	0.005	-0.824	-0.215	-0.694	-0.452*	0.003	-0.670	-0.164	-0.488
DCA Ring 5 – CT	0.405**	0.077	-0.046	0.719	0.429	0.205*	0.204	-0.114	0486	0.208

*Pearson's Correlation **Spearman's rho Correlation

Table S8: Ring Correlation of ICA gfmfERG with AXL & CT in 3min RLRT among myopes & non-myopes

Ring Wise Correlation with AXL & CT	Myope					Non-Myope				
	r-value	p-value	95% CI		Effect Size	r-value	p-value	95% CI		Effect Size
			Lower	Upper				Lower	Upper	
ICA Ring 1 – AXL	0.045*	0.851	-0.406	0.478	0.045	-0.310**	0.184	-0.661	0.154	-0.320
ICA Ring 1 – CT	-0.100*	0.675	-0.520	0.358	-0.100	0.028**	0.906	-0.419	0.465	0.028
ICA Ring 2 – AXL	-0.438*	0.053	-0.738	0.005	-0470	0.341**	0.141	-0.120	0.681	0.355
ICA Ring 2 – CT	0.282*	0.229	-0.184	0.644	0.289	-0.165**	0.488	-0.566	0.300	-0.166
ICA Ring 3 – AXL	-0.376*	0.102	-0.702	0.080	-0.395	0.243**	0.301	-0.223	0.619	0.248
ICA Ring 3 – CT	0.341*	0.142	-0.120	0.681	0.355	-0.415**	0.069	-0.724	0.034	-0.441
ICA Ring 4 – AXL	-0.534*	0.015	-0.790	-0.119	-0.595	0.614**	0.004	0.235	0.831	0.715
ICA Ring 4 – CT	0.425*	0.062	-0.022	0.730	0.453	-0.368**	0.111	-0.697	0.089	-0.386
ICA Ring 5 – AXL	-0.434*	0.056	-0.735	0.010	-0.465	-0.433**	0.005	-0.656	-0.141	-0.464
ICA Ring 5 – CT	0.384*	0.094	-0.070	0.707	0.405	0.159**	0.327	-0.160	0.448	0.161

*Pearson's Correlation **Spearman's rho Correlation

Table S9: Ring Correlation of DCT gfmfERG with AXL & CT in 1min RLRT among myopes & non-myopes

Ring Wise Correlation with AXL & CT	Myope					Non-Myope				
	r-value	p-value	95% CI		Effect Size	r-value	p-value	95% CI		Effect Size
			Lower	Upper				Lower	Upper	
DCT Ring 1 – AXL	0.036**	0.904	-0.505	0.556	0.036	0.071**	0.780	-0.410	0.520	0.071
DCT Ring 1 – CT	-0.220**	0.450	-0.672	0.352	-0.223	0.229**	0.360	-0.266	0.629	0.234
DCT Ring 2 – AXL	0.188**	0.520	-0.381	0.653	0.190	0.430**	0.075	-0.047	0.747	0.459
DCT Ring 2 – CT	-0.553**	0.040	-0.838	-0.032	-0.623	0.207**	0.411	-0.288	0.614	0.210
DCT Ring 3 – AXL	0.289*	0.317	-0.286	0.710	0.297	0.520**	0.027	0.070	0.794	0.576
DCT Ring 3 – CT	-0.270*	0.350	-0.700	0.304	-0.277	0.222**	0.375	-0.273	0.624	0.226
DCT Ring 4 – AXL	0.546*	0.043	0.022	0.835	0.613	0.417*	0.085	-0.062	0.740	0.444
DCT Ring 4 – CT	-0.479*	0.083	-0.805	0.069	-0.522	-0.030*	0.905	-0.490	0.443	-0.030
DCT Ring 5 – AXL	0.543*	0.045	0.018	0.834	0.609	0.411*	0.090	-0.069	0.737	0.437
DCT Ring 5 – CT	-0.723*	0.003	-0.906	-0.313	-0.915	0.177*	0.483	-0.316	0.595	0.179

*Pearson's Correlation **Spearman's rho Correlation

STRICTLY CONFIDENTIAL

Table S10: Ring Correlation of ICT gfmfERG with AXL & CT in 1min RLRT among myopes & non-myopes

Ring Wise Correlation with AXL & CT	Myope					Non-Myope				
	r-value	p-value	95% CI		Effect Size	r-value	p-value	95% CI		Effect Size
			Lower	Upper				Lower	Upper	
ICT Ring 1 – AXL	0.027*	0.928	-0.511	0.549	0.027	-0.148*	0.557	-0.575	0.342	-0.149
ICT Ring 1 – CT	-0.148*	0.613	-0.629	0.415	-0.149	-0.356*	0.147	-0.706	0.133	-0.372
ICT Ring 2 – AXL	0.274*	0.343	-0.300	0.703	0.281	0.075*	0.767	-0.406	0.524	0.075
ICT Ring 2 – CT	-0.143*	0.626	-0.626	0.419	-0.144	-0.148*	0.559	-0.575	0.343	-0.149
ICT Ring 3 – AXL	0.037*	0.901	-0.504	0.556	0.037	0.025*	0.920	-0.447	0.487	0.025
ICT Ring 3 – CT	-0.251*	0.386	-0.690	0.322	-0.257	-0.290*	0.241	-0.667	0.203	-0.300
ICT Ring 4 – AXL	-0.117*	0.691	-0.610	0.441	-0.117	0.117*	0.645	-0.371	0.553	0.117
ICT Ring 4 – CT	-0.065*	0.825	-0.576	0.482	-0.065	0.382*	0.118	-0.103	0.754	0.403
ICT Ring 5 – AXL	-0.115*	0.695	-0.609	0.443	-0.116	-0.290**	0.243	-0.667	0.205	-0.298
ICT Ring 5 – CT	-0.249*	0.391	-0.688	-0.718	0.272	-0.006**	0.980	-0.472	0.462	-0.006

*Pearson's Correlation **Spearman's rho Correlation

Table S11: Ring Correlation of DCA gfmfERG with AXL & CT in 1min RLRT among myopes & non-myopes

Ring Wise Correlation with AXL & CT	Myope					Non-Myope				
	r-value	p-value	95% CI		Effect Size	r-value	p-value	95% CI		Effect Size
			Lower	Upper				Lower	Upper	
DCA Ring 1 – AXL	0.035**	0.905	-0.505	0.555	0.035	-0.294*	0.236	-0.669	0.200	-0.303
DCA Ring 1 – CT	-0.235**	0.417	-0.681	0.338	-0.240	0.069*	0.785	-0.411	0.519	0.069
DCA Ring 2 – AXL	-0.096**	0.745	-0.596	0.458	-0.096	-0.142*	0.573	0.348	-0.143	0.258
DCA Ring 2 – CT	-0.154**	0.599	-0.633	0.410	-0.155	0.342*	0.165	-0.148	0.698	0.258
DCA Ring 3 – AXL	-0.509**	0.063	-0.818	0.030	-0.561	-0.250**	0.318	-0.642	0.246	-0.255
DCA Ring 3 – CT	0.024**	0.934	-0.513	0.548	0.024	0.048**	0.850	-0.428	0.504	0.048
DCA Ring 4 – AXL	-0.522**	0.056	-0.824	0.012	-0.579	0.163*	0.517	-0.329	0.586	0.165
DCA Ring 4 – CT	0.095**	0.748	-0.459	0.595	0.095	0.172*	0.494	-0.320	0.592	0.174
DCA Ring 5 – AXL	-0.479**	0.083	-0.805	0.070	-0.521	0.020**	0.937	-0.451	0.483	0.020
DCA Ring 5 – CT	0.430**	0.125	-0.131	0.782	0.459	-0.091**	0.719	-0.535	0.392	-0.091

*Pearson's Correlation **Spearman's rho Correlation

Table S12: Ring Correlation of ICA gfmfERG with AXL & CT in 1min RLRT among myopes & non-myopes

Ring Wise Correlation with AXL & CT	Myope					Non-Myope				
	r-value	p-value	95% CI		Effect Size	r-value	p-value	95% CI		Effect Size
			Lower	Upper				Lower	Upper	
ICA Ring 1 – AXL	-0.308**	0.283	-0.721	0.266	-0.319	-0.450**	0.061	-0.758	0.022	-0.484
ICA Ring 1 – CT	0.506**	0.065	-0.034	0.817	0.557	-0.277**	0.266	-0.659	0.218	-0.284
ICA Ring 2 – AXL	-0.267**	0.357	-0.698	0.308	-0.273	0.080*	0.752	-0.402	0.527	0.080
ICA Ring 2 – CT	-0.103**	0.725	-0.601	0.452	-0.104	0.303*	0.221	-0.190	0.675	0.313
ICA Ring 3 – AXL	-0.529**	0.052	-0.827	0.002	-0.589	0.183*	0.468	-0.311	0.599	0.185
ICA Ring 3 – CT	-0.258**	0.374	-0.693	0.316	-0.264	-0.079*	0.755	-0.527	0.403	-0.079
ICA Ring 4 – AXL	-0.834**	<0.001	-0.946	-0.544	-1.201	0.197*	0.434	-0.297	0.608	0.199
ICA Ring 4 – CT	0.206**	0.481	-0.365	0.664	0.208	-0.126*	0.618	-0.560	0.362	-0.127
ICA Ring 5 – AXL	-0.680**	0.007	-0.890	-0.234	-0.829	0.136*	0.590	-0.353	0.567	0.137
ICA Ring 5 – CT	0.456**	0.101	-0.098	0.795	0.493	-0.030*	0.905	-0.490	0.443	-0.030

*Pearson's Correlation **Spearman's rho Correlation

STRICTLY CONFIDENTIAL
